# Supplementary material for: Effects of Vibrant Soundbridge on tinnitus accompanied by sensorineural hearing loss
Source: PLoS One. 2020 Feb 4;15(2):e0228498. doi: 10.1371/journal.pone.0228498 (PMC6999863; doi:10.1371/journal.pone.0228498)
Supplement: Attachment — Submitted filename: rebuttal_letter.docx [file pone.0228498.s002.docx]

**Academic Editor:** The appropriate statistical tests (and corrections included where appropriate) need to be applied and reported, the reporting of statistical results needs to follow standard protocols.

- Thank you for valuable comments. We have obtained professional statistical support and have applied the appropriate statistical tests and reported the results using standard protocols.

- Firstly, we have provided a new Table 2, instead of Figures 1 and 2. While previous figures contained only *p*-values, the current Table 2 now contains the raw values (mean and standard deviations), *t*-values, as well as probability values. We have also reported t-values in the manuscript. We believe that the current format of reporting would be more helpful for understanding the data and the analysis.

- Secondly, we have provided Table 3 instead of Figure 3. The current Table 3 and Figure 3 describe “influencing factors” using multiple regression analysis. In the current Table 3, we showed all the raw values of the variables with unstandardized regression coefficients and standard errors. Previously reported R-squared and p-values are also contained in the current table. This table clearly shows the results in detail, so that the readers can easily understand the analysis method used and how the analysis model fits our study. Relevant values are also reported in the manuscript.

- Please see the highlighted sentences in the manuscript.
