# Supplementary material for: Effects of Vibrant Soundbridge on tinnitus accompanied by sensorineural hearing loss
Source: PLoS One. 2020 Feb 4;15(2):e0228498. doi: 10.1371/journal.pone.0228498 (PMC6999863; doi:10.1371/journal.pone.0228498)
Supplement: Attachment — Submitted filename: Response to Reviewers.docx [file pone.0228498.s003.docx]

**Reviewer #1:**

1. Page 2, lines 24-29: Instead of showing p-values, I suggest to depict point estimates and their 95% confidence intervals.

- Thank you for your valuable suggestion. We have reanalyzed the values and modified the paragraph as recommended.

- (lines 24-31) “*VAS scores for loudness (mean difference: 1.9, 95% CI: 0.6, 3.1), awareness (mean difference: 1.6, 95% CI: 0.4, 2.8), and annoyance (mean difference: 1.7, 95% CI: 0.7, 2.8) showed significant improvements from baseline to 12 months after surgery. In addition, THI scores showed a significant decrease (mean difference: 13.8, 95% CI: 2.9, 24.9). The average hearing threshold level, WRS, and most comfortable level (MCL) also showed significant improvements at 12 months after surgery (mean difference: 17.3, 95% CI: 13.3, 21.3; mean difference: −7.6, 95% CI: −15.1, −0.1; mean difference: 26.3, 95% CI: 22.9, 29.6, respectively). Among the aforementioned factors, changes in MCL were best correlated with those in THI scores (mean difference: 2.55, 95% CI: 0.90, 4.21).”*

2. Page 5, lines 91-93: I was curious how many subjects were not included because of the lack of follow-up, audiological tests, and/or self-assessment questionnaires. Inclusion criteria #1-#3 and exclusion criteria #1-#3 would provide the number of study subjects within a 7-month period.

- Thank you for your question. We retrospectively reviewed data for patients who received VSB between January 2016 and July 2019. At the first medical examination, we routinely asked the patients about the presence and duration of tinnitus. Although we did not observe the patients for 2 years, the durations (>2 years) were self-reported by the patients. We agree than the current information can cause confusion and have made the relevant changes in the revised manuscript.

*- (lines 92-93) “…3) stable chronic tinnitus that did not respond to any previous treatments,…”*

*- (lines 98-101)* “*In total, 47 patients underwent VSB implantation between January 2016 and July 2019. From these, 3 patients were excluded because they had mixed hearing loss. In addition, 16 patients who did not experience tinnitus, 4 who were followed up for less than 1 year, 7 who did not complete the questionnaires, and 1 foreigner for whom a speech discrimination test was not available were excluded.”*

3. Table 2:

(3a) Column "A-B": I assumed the test for A-B comparison was a paired t test. For paired t tests, I would suggest to replace the percent change with a standard deviation of the difference. The percent change can be easily calculated by readers if necessary.

(3b) Column "Probability": I suggest to replace "probability" with 'p-value'.

- Thank you for the constructive advice. We have amended the table as per your recommendations.

*- Table 2. Comparing means of preoperative unaided and postoperative aided ear conditions (N = 16).*

*THI = tinnitus handicap inventory, VAS = visual analog scale, PTA = pure-tone average, WRS = word recognition score, MCL = most comfortable level, M = mean, SD = standard deviation, CI =confidence interval*

4. Page 10, lines 189-198: With the sample size of 16, the regression analyses were likely to be under power, meaning that the only significant finding might not be reproducible (i.e., high type 2 error) or falsely observed (i.e., high type 1 error). Please discuss this as a limitation in the Discussion section.

- Thank you for your valuable comments. We agree that the sample size was too small to perform regression analysis and may have resulted in type II errors. We have mentioned this in the Discussion section and added it as a limitation.

*- (lines 255-258) “**Finally, a sample size compromises the power for the detection of treatment effects and increases the possibility of false-negative results. Thus, the small sample size in this study may be a reason behind some of the insignificant results.”*

*- (lines 272-274) “Furthermore, the sample size was small and may have resulted in type II errors, as discussed earlier. Future studies should also include larger samples to overcome this limitation.”*

5. Table 3: I suggest to replace the standard errors with 95% confidence intervals for the regression estimates.

- Thank you for the constructive advice. We have reanalyzed the values and replaced the standard errors with 95% confidence intervals in Table 3.

*-* *Table 3. Multiple regression analysis for the association between audiological improvement and tinnitus improvement (N = 16).*

| Variables | ΔTHI | ΔVAS for loudness | ΔVAS for awareness | ΔVAS for annoyance |
| --- | --- | --- | --- | --- |
| ΔPTA | -0.41  (-1.88, 1.05) | -0.09  (-0.33, 0.15) | -0.11  (-0.33, 0.12) | -0.13  (-0.33, 0.08) |
| ΔWRS | 0.28  (-0.37, 0.94) | 0.03  (-0.14, 0.08) | 0.02  (-0.13, 0.08) | 0.02  (-0.12, 0.07) |
| ΔMCL | 2.55**  (0.90, 4.21) | 0.16  (-0.12, 0.43) | 0.19  (-0.06, 0.45) | 0.17  (-0.07, 0.40) |
| R-squared | 0.60 | 0.12 | 0.18 | 0.18 |

*THI = tinnitus handicap inventory, VAS = visual analog scale, PTA = pure-tone average, WRS = word recognition score, MCL = most comfortable level, M = mean, SD = standard deviation*

*Unstandardized regression coefficients are shown, and 95% confidence intervals are given in parentheses.*

*2-tailed test; **p < 0.01*

**Reviewer #2:** This is a well-written manuscript that should be of interest to some of the journal's readers. I have just a few minor comments:

1. Page 4, Lines 79-80: "Thus, we hypothesized that VSB . . . " This sentence needs to be re-worded.

- Thank you for the positive feedback and comments. We have rephrased the sentence in the revised manuscript.

- (lines 81-82) “*Accordingly, we hypothesized that the use of VSB as a hearing device would have a beneficial effect on tinnitus in patients with sensorineural hearing loss.*”

2. Table 2: add units for PTA and MCL (e.g., dB HL)

- Thank you for pointing this out. We have added units for PTA, WRS, and MCL in Table 2.

3. Page 9, Line 170: "The questionnaire results demonstrated statistically significant improvements . . ." Add the word "statistically"

4. Page 9, Lines 173-174: "Based on the 20% criterion, 7 patients . . . " add "20% criterion"

- Thank you for the valuable suggestions. We have modified the sentences as recommended.

- (lines 173-174) “*The questionnaire results demonstrated statistically significant improvements after VSB implantation.”*

- (lines 176-178) “*Based on the 20% criterion, 7 patients (43.8%) reported improvement in tinnitus loudness, 8 patients (50.0%) reported no change, and 1 patient (6.2%) reported louder tinnitus after implantation.”*

5. Page 10, Line 194: "only increased MCL was significantly . . ." I think the authors mean "decreased MCL"

- We apologize for the overlook. We have rectified the error in the revised manuscript.

- (lines 196-198) “*When we performed multiple regression analysis, only decreased MCL was significantly and positively related to the improvement of THI.”*

6. Page 11, Lines 213-214: "There are no known pathophysiological accounts pertaining to . . ." This sentence should be re-worded because I'm not sure what "pathophysiological accounts" means.

- We apologize for the lack of clarity. We have rephrased the sentence and merged it with the subsequent sentence in the revised manuscript.

- (lines 219-221) “*The mechanism underlying the beneficial effect of VSB on tinnitus remains unclear, although it may be similar to that of other hearing devices.*”

7. Page 12, Lines 249-251: The last 2 sentences should be deleted because the point of this VAS was NOT to compare current conditions with the patient's memory from 1 year ago. The goal of this VAS was to assess the patient's condition at the time they answered the question: once pre-surgery, and once post-surgery.

- Thank you for the suggestion. We have deleted the sentences from the revised manuscript.

8. Page 13, Line 261: "relief from hearing gain" Do the authors mean "relief from hearing loss"?

- We apologize for the lack of clarity. Because the pathophysiology of tinnitus includes psychological aspects, reducing emotional stress is crucial for managing the condition tinnitus. Emotional stress caused by hearing loss also affects the severity of tinnitus, and hearing gain by means of hearing devices can help in overcoming this emotional stress. Accordingly, we used the expression “relief from hearing gain”, although we agree that the phrase is not clear. We have now modified the sentence in the revised manuscript.

- (lines 264-266) “*Although tinnitus retraining therapy or direct counseling for tinnitus were not performed in the present study, a frequent fitting schedule with counseling and stress reduction as a consequence of hearing gain would qualify as combined management.”*
